# Supplementary material for: A compact time-domain diffuse optical tomography system for cortical neuroimaging
Source: Imaging Neurosci (Camb). 2025 Feb 20;3:imag_a_00475. doi: 10.1162/imag_a_00475 (PMC12319781; doi:10.1162/imag_a_00475)
Supplement: Supplementary Material [file imag_a_00475-supp.pdf]

# Supplementary Material

Mathematical Appendix. Derivation of the Jacobian for log(sum), mean and variance moments from the time-resolved Jacobian.

Below we derive the Jacobian for the log(sum), mean and variance moments of the TPSF. This derivation uses the Leibniz rules for the derivative of a product (eq. 1) and the derivative of a quotient (eq. 2).

With the following notation: $\frac{\partial x}{\partial \mu}$: $d(x)$

We have:

(1) $d(xy) \ = \ yd(x) \ + \ xd(y)$

(2) $d(\frac{x}{y}) \ = \ \frac{y * d(x) - x * d(y)}{y^2}$

We derive the Jacobian ($J_M = \frac{\partial M}{\partial \mu}$) for each of the following data types, where μ denotes an optical property, as a function of the time-resolved Jacobian $J_H$

- sum moment $M0_H \ = \ \Sigma H$, where $H$ corresponds to the model TPSF, and the Σ is over time bins *t*

$$J_{M0_H} = \frac{\partial\, M0_H}{\partial \mu} = \frac{\partial\, \Sigma\, H}{\partial \mu} \ = \Sigma J_H$$

It can be seen that $J_{M0_H}$ is dependent on the scale of the data. We can normalize it by the total counts $M0_H$ to get a scale independent Jacobian. We'll call that normalized Jacobian $J_{M0_H}*$

$$J_{M0_H}* = \frac{J_{M0_H}}{M0_H}$$

This is the logarithmic derivative of $M0_H$, or the Jacobian of the log sum moment. The log sum moment is also known as the optical density. Therefore

$$J_{log(M0_H)} = \ \Sigma J_H$$

- mean moment $M1_H = \frac{\Sigma (H * t)}{M0_H}$

$$J_{M1_H} = \frac{\partial M1_H}{\partial \mu} = \frac{\partial \frac{\Sigma (H * t)}{M0_H}}{\partial \mu} = \frac{(M0_H * \Sigma \frac{\partial H}{\partial \mu} t) - (\Sigma (H * t) * \frac{\partial M0_H}{\partial \mu})}{M0_H^{\ 2}},$$

therefore

$$J_{M1_H} = \frac{\Sigma(J_H * t)}{M0_H} - \frac{M1_H * \Sigma J_H}{M0_H}$$

- Variance moment $M2_H = \frac{\Sigma ((t - M1_H)^2 * H)}{M0_H}$

$$J_{M2_H} = \frac{\partial M2_H}{\partial \mu} = \frac{\partial \frac{\Sigma ((t - M1_H)^2 * H)}{M0_H}}{\partial \mu} = \frac{( M0_H * d(\Sigma (t - M1_H)^2 * H ) ) - ( \Sigma (t - M1_H)^2 * H * d(M0_H))}{M0_H^{\ 2}}$$

$$J_{M2_H} = \frac{d(\Sigma ((t - M1_H)^2 * H) )}{M0_H} - \frac{M2_H * \Sigma J_H}{M0_H} = \frac{\Sigma ( -2 * (t - M1_H) d(M1_H) * H + d(H) * (t - M1_H)^2 )}{M0_H} - \frac{M2_H * \Sigma J_H}{M0_H}$$

$$J_{M2_H} = \frac{\Sigma ( -2 * (t - M1_H) J_{M1_H} * H + J_H * (t - M1_H)^2 )}{M0_H} - \frac{M2_H * \Sigma J_H}{M0_H}$$

$$J_{M2_H} = \frac{-2 * J_{M1_H} \Sigma ( (t - M1_H) * H )}{M0_H} + \frac{\Sigma( J_H * (t - M1_H)^2 )}{M0_H} - \frac{M2_H * \Sigma J_H}{M0_H}$$

$$J_{M2_H} = \frac{-2 * J_{M1_H} (\Sigma ( H * t) - M1_H \Sigma H ))}{M0_H} + \frac{\Sigma( J_H * (t - M1_H)^2 )}{M0_H} - \frac{M2_H * \Sigma J_H}{M0_H}$$

$$J_{M2_H} = -2 * J_{M1_H} (\frac{\Sigma ( H * t )}{M0_H} - \frac{M1_H \Sigma H )}{M0_H}) + \frac{\Sigma( J_H * (t - M1_H)^2 )}{M0_H} - \frac{M2_H * \Sigma J_H}{M0_H}$$

$$J_{M2_H} = -2 * J_{M1_H} (M1_H - \frac{M1_H M0_H )}{M0_H}) + \frac{\Sigma( J_H * (t - M1_H)^2 )}{M0_H} - \frac{M2_H * \Sigma J_H}{M0_H}$$

$$J_{M2_H} = -2 * J_{M1_H} (M1_H - M1_H) + \frac{\Sigma( J_H * (t - M1_H)^2 )}{M0_H} - \frac{M2_H * \Sigma J_H}{M0_H},$$

therefore

$$J_{M2_H} = \frac{\Sigma(\ J_H * \ (t - M1_H)^2\ )}{M0_H} - \frac{M2_H * \Sigma J_H}{M0_H}$$

These quantities can be computed on the fly from the time resolved Jacobian $J_H$ and corresponding model TPSFs $H$.

| Region | Oxyhemoglobin (mM) | Deoxyhemoglobin (mM) | Scatter power | Scatter amplitude |
|---|---|---|---|---|
| Skin | 0.057 | 0.031 | 1.16 | 0.53 |
| Skull | 0.044 | 0.019 | 0.89 | 0.73 |
| CSF | 0.011 | 0.008 | 0 | 0.3 |
| Gray matter | 0.056 | 0.035 | 1.74 | 0.51 |
| White matter | 0.068 | 0.027 | 1.31 | 0.82 |

Table S1. Baseline optical properties of the head model. Reproduced from (Doulgerakis et al., 2019).

| | Flow1 | Flow2 | Delta |
|---|---|---|---|
| **Detector Type** | Silicon | Silicon | = |
| **Responsivity** | | | |
| 690nm | $0.72\times10^{-8}$ m$^2$ sr | $1.0\times10^{-8}$ m$^2$ sr | +38% |
| 850nm/905nm | $0.25\times10^{-8}$ m$^2$ sr | $0.25\times10^{-8}$ m$^2$ sr | = |
| **DNL** | 0.5 | 0.15 | -70% |
| **IRF FWHM / 10% / 1%** | | | |
| 690nm | 290ps / 780ps / 1620ps | 285ps / 710ps / 1740ps | -2% / -9% / +7% |
| 850nm/905nm | 350ps / 1000ps / 2150ps | 305ps / 725ps / 1715ps | -13% / -27% / -20% |
| **IRF Stability** | | | |
| FWHM Variability | < ±10ps | < ±10ps | = |
| Mean ToF Variability | < ±10ps | <±2ps | - 80% |
| Warm-up Time | 30 minutes | 20 minutes | - 33% |
| **Afterpulsing** | | | |
| 690nm | 0.3% | 0.3% | = |
| 850nm/905nm | 0.5% | 0.2% | -60% |
| **Max Count Rate** | 1500 Mcps | 2200 Mcps | + 46% |
| **Dynamic Range** | 4-5 Orders of Magnitude | 5-6 Orders of Magnitude | +900% |
| **MEDPHOT Correlation** | | | |
| 690nm | 0.988 | 0.992 | = |
| 850nm/905nm | 0.993 | 0.988 | = |
| **MEDPHOT Uncertainty** | +/-2.5% | +/-1% | -60% |
| **Maximum # of Channels** | 2200 | 3500 | +59% |
| **Power per Channel** | 25mW | 13mW | -48% |

Table S2. Comparison of key performance metrics between Flow1 and Flow2.

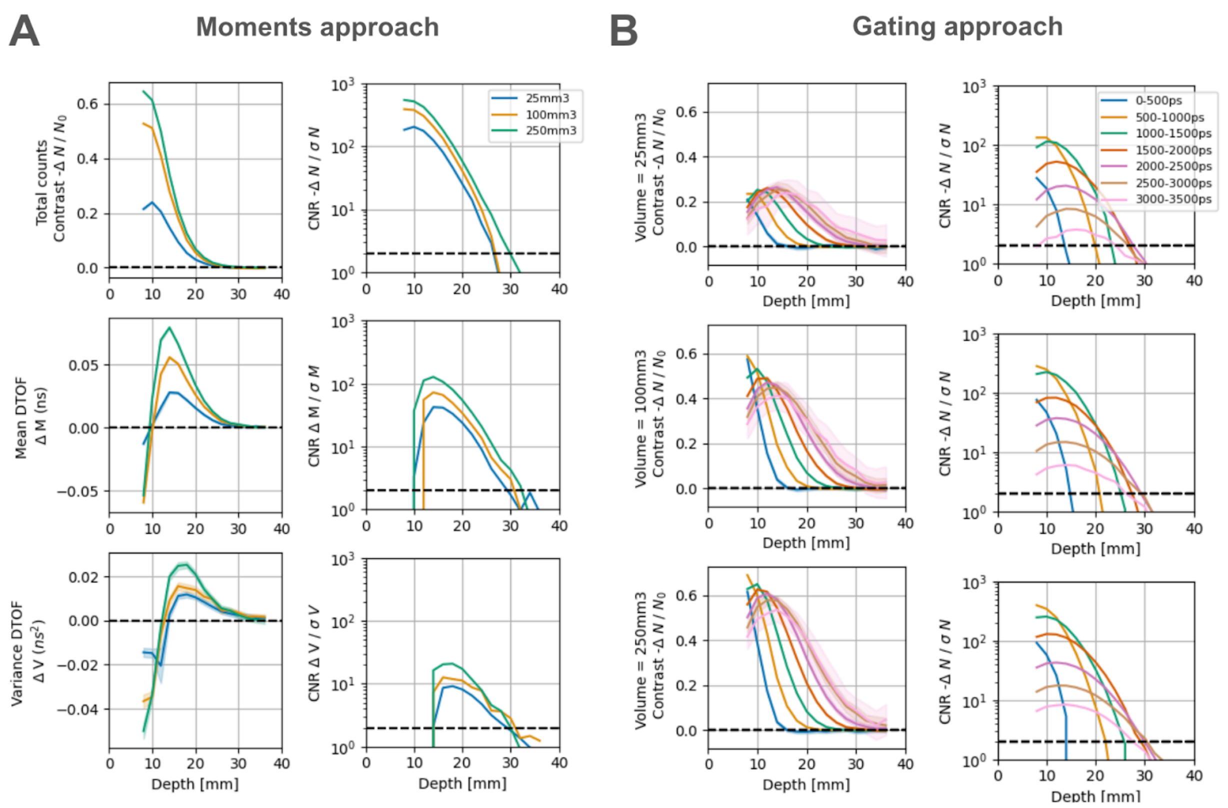


**Fig. S1. nEUROPt protocol results for 690 nm wavelength.**
**(A)** Left) Contrast as a function of depth for three different moments of DTOFs (sum, mean and variance are the three rows respectively). Each color represents the volume of a different PVC cylinder used in the experiment. Right) Same as (left) but the contrast-to-noise ratio (CNR) at different depths. **(B)** Contrast (left) and CNR (right) are shown as a function of depth for the three different PVC cylinders used (each row). Each color corresponds to different time gates used for analysis. Both A and B are results with the 690 nm wavelength and a representative channel. For the results with 905 nm wavelength see Fig. 5.

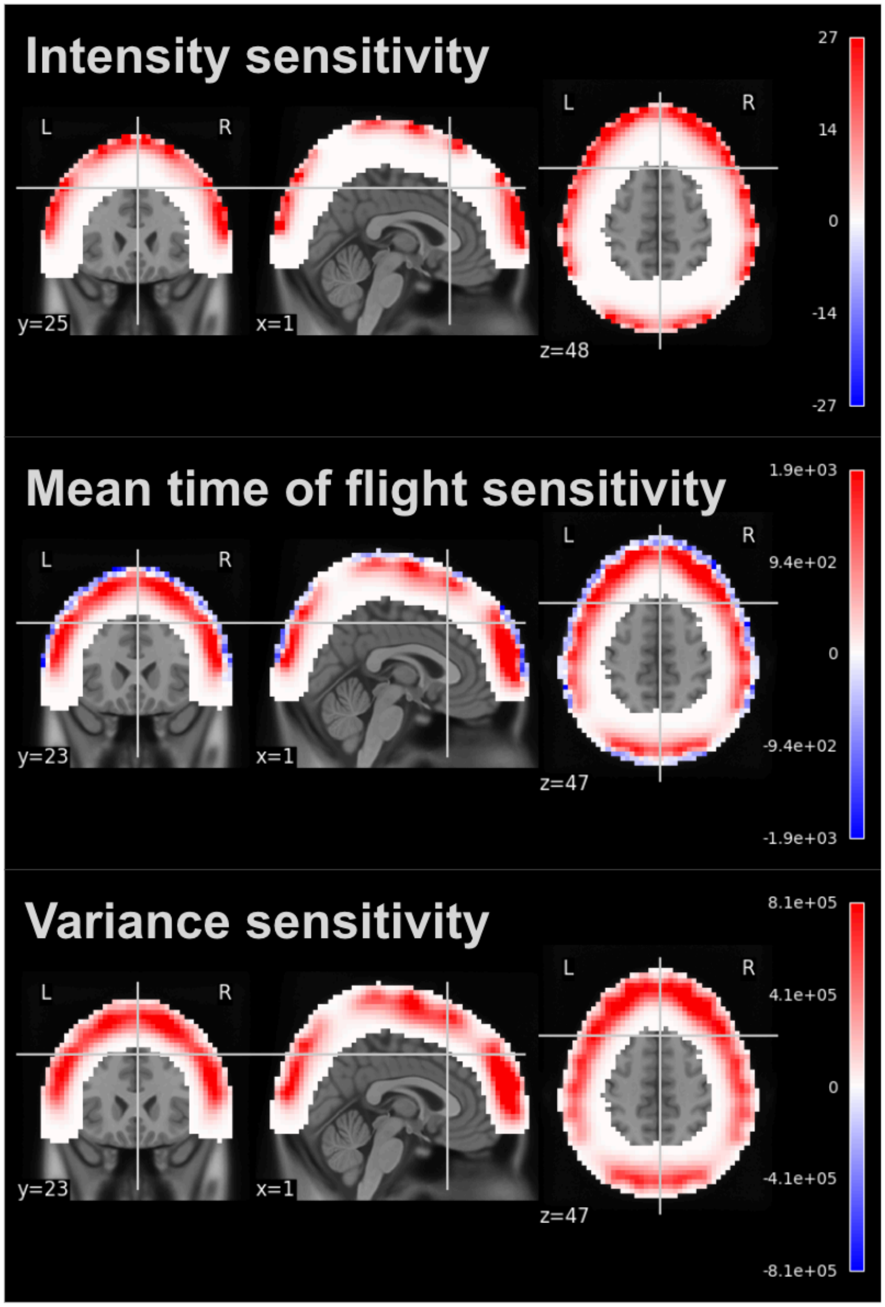


**Fig. S2. Sensitivity profiles for intensity, mean time of flight, and variance moments.** The computed Jacobians are shown for the first three moments (each row) in the MNI space using the coronal (left), sagittal (middle), and axial (right) views. Note that higher moments exhibit sensitivity to deeper layers as indicated by the thicker red regions. The areas with lower sensitivity in the superficial layers are due to the gaps between the headset plates and lower number of channels in those regions (e.g., the area between the prefrontal and frontoparietal plate).
